# Unified InterPlanetary Smart Parking Network for Maximum End-User Flexibility

**DOI:** 10.3390/s22010221

**Published:** 2021-12-29

**Authors:** Ciprian Iacobescu, Gabriel Oltean, Camelia Florea, Bogdan Burtea

**Affiliations:** 1 Bases of Electronics Department, Technical University of Cluj-Napoca, 400027 Cluj-Napoca, Romania; Gabriel.Oltean@bel.utcluj.ro (G.O.); burtea.n.bogdan@campus.utcluj.ro (B.B.); 2 Communications Department, Technical University of Cluj-Napoca, 400027 Cluj-Napoca, Romania

**Keywords:** protocol, smart parking, federated model, IPFS, distributed systems, collaborative smart parking

## Abstract

Technological breakthroughs have offered innovative solutions for smart parking systems, independent of the use of computer vision, smart sensors, gap sensing, and other variations. We now have a high degree of confidence in spot classification or object detection at the parking level. The only thing missing is end-user satisfaction, as users are forced to use multiple interfaces to find a parking spot in a geographical area. We propose a trustless federated model that will add a layer of abstraction between the technology and the human interface to facilitate user adoption and responsible data acquisition by leveraging a federated identity protocol based on Zero Knowledge Cryptography. No central authority is needed for the model to work; thus, it is trustless. Chained trust relationships generate a graph of trustworthiness, which is necessary to bridge the gap from one smart parking program to an intelligent system that enables smart cities. With the help of Zero Knowledge Cryptography, end users can attain a high degree of mobility and anonymity while using a diverse array of service providers. From an investor’s standpoint, the usage of IPFS (InterPlanetary File System) lowers operational costs, increases service resilience, and decentralizes the network of smart parking solutions. A peer-to-peer content addressing system ensures that the data are moved close to the users without deploying expensive cloud-based infrastructure. The result is a macro system with independent actors that feed each other data and expose information in a common protocol. Different client implementations can offer the same experience, even though the parking providers use different technologies. We call this InterPlanetary Smart Parking Architecture NOW—IPSPAN.

## 1. Introduction

Recent advancements in remote sensors, low-powered processing devices, autonomous vehicles, and particularly communication technologies, such as 5G, have improved the possibility of transforming the concept of a smart city into reality. Smart parking solutions have been implemented as pilot projects or in small numbers due to high implementation costs. Even in places where smart parking has been deployed at a large parking place, user adoption or satisfaction is low, and so is the impact of the project. 

Multiple parking providers might offer smart parking solutions using different technologies in a particular geographical area. Most implementations are centralized. Each service provider has its infrastructure, uses its own application interface model, and a custom user interface in a web page or mobile application. At the city level, the end-user can hope to receive partial information by using multiple interfaces. Citizens want to have meaningful information at their fingertips (smart devices, smartphones, car’s navigation, etc.) in a unified manner. Urban areas often have a shortage in parking places and optimal allocation problems. Some estimates say that 30 percent of traffic in rush hour is due to people looking for a vacant spot [1]. 

Researchers in the field estimate that a driver takes between 4 and 15 min searching for a place to park [2]. This may lead to increases in stress levels, air pollution, and accidents. Despite municipalities encouraging alternative means of transportation (bicycles, e-scouters, etc.) or public transport (buses, trams, and metro), people will use personal cars for convenience. An urban area filled with sensors, data collection, data centers, etc., is a city with sensors, not a Smart City. It is a vastly different concept, although they are often mixed.

Collecting data, analyzing it, and making decisions based on it are insufficient. Smart cities must allow bidirectional communication through ICT (information and communications technology) between the citizens, the services in the city, and the authorities. At the same time, reliable data are needed to optimize algorithms. The same principles apply to smart parking, which must be considered as a component of the smart city concept. 

A solution for smart cities that solves the problem of finding a free parking spot was developed by our team at Parking Spotter [3]. Our software can identify the empty spots from a parking lot or on the side of the road. Each parking spot is mapped on the frames from the CCTV cameras using our Configuration Tool. The statuses of the parking spots predicted by our algorithm are sent to a cloud server. The information from the cloud server is available for drivers in a mobile application and parking owners in a dashboard, along with reports and different statistics.

Assuming the number of parking spots and cars remains unchanged, a system that offers drivers near real-time information of parking availability could present the opportunity of better planning a trip so that time spent cruising in urban centers is reduced to a minimum. 

**Figure 1:**
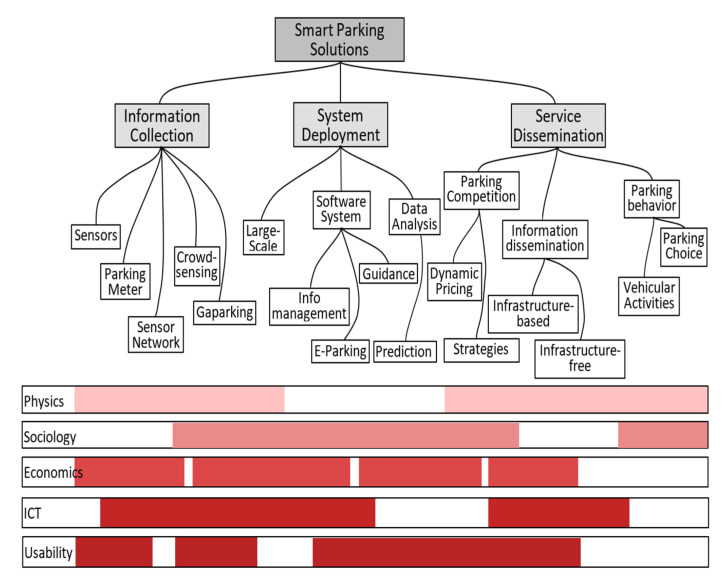
Categories of smart parking solutions and the spectrum of disciplines related to the topic, Source: A Survey of Smart Parking Solutions [4].

From investor’s perspective, as can be observed in Figure 1, a smart parking solution is viable if three main areas are sustainable: physics, economics, and ICT. End-user satisfaction can be extrapolated by the overlapping of sociology and usability. Unfortunately, there is not much common ground between parking providers (private or public investors) and end-users.

Trista Lin, Hervé Rivano, and Frédéric Le Mouël et al. [4] analyzed the literature from 2000 to 2016 on parking solutions and proposed three macro-themes: information collection, system deployment, and service dissemination (see Figure 1). The study states that, although there are many deployment projects and apps, only very few drivers with SFpark (San Francisco) and LA Express Park (Los Angeles) could benefit from smart parking. Even though the initiative was good, the authors give the city of Nice smart parking as a failed project and identify the following issues:

- Sensors are not robust—they can malfunction and do not always have health monitoring functions.
- Remote sensors communicate at a low periodicity due to battery life concerns; sometimes the radio signal might be blocked by a car occupying the parking spot.
- Drivers do not receive information based on GPS location—information is broadcasted about the status of the entire system, which has a slowdown effect on user mobile devices.

### 1.1. The Current Model and Traditional Implementations

At the time of writing, various smart parking systems can be found in the literature, focusing primarily on parking live availability. Innovation in parking solutions is manifested in many forms: video analytics, smart sensors, gap parking, barrier parking, etc. There also are public parking lots administered by municipalities and private ones opened to the public. Not all the implementations have a mobile app; most of those who have an app show only the parking areas under their administration. 

Innovation has hit the market but missed the end-user as each implementation approach tries to dominate the bubble it operates. While searching for available parking, the user is forced to have multiple mobile applications. An aggregation service might improve the experience for the end-user, but the Parking Providers will lose autonomy. The degree of centralization will increase, and a single point of failure for the whole system will be created.

**Figure 2:**
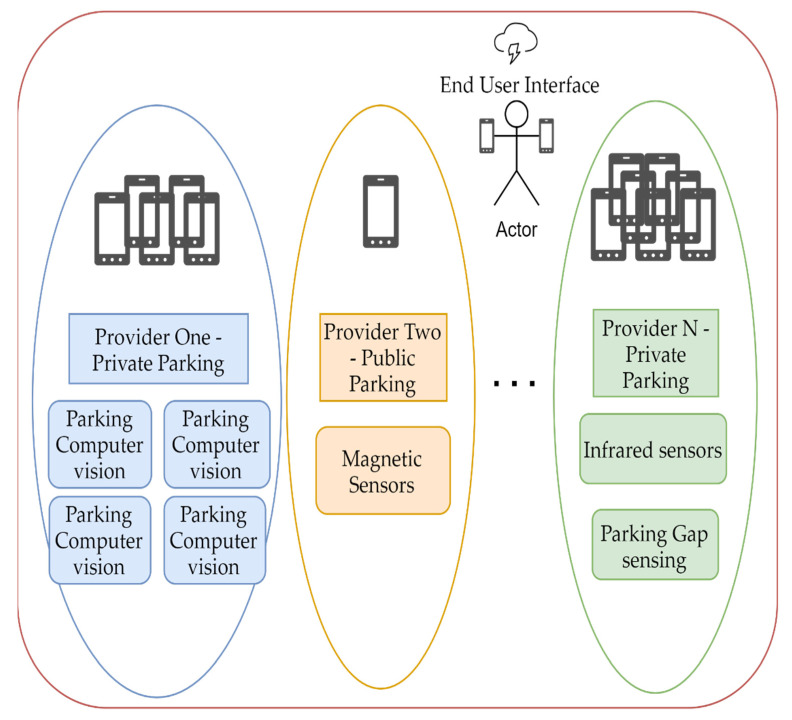
The current model of smart parking implementations in a certain geographical area, covered by three independent parking providers.

Different parking providers might use similar technology but not let users interact with other service providers. Presume parking provider two from Figure 2 is a municipal entity with all public parking bays under its administration and citizens can interact with their service using a simple mobile app. A commercial entity, like provider one, would find it costly to integrate with other service providers on a one-to-one basis. The end-user is stuck in each mobile app bubble and needs to install multiple applications for the same processes. As seen in Table 1, technologies vary from application to application, and very few solution providers implemented more than one technology. 

According to the study presented in [5], the researchers identified the following mobile applications and their use of technologies and sensors.

Smart parking solutions have been attracting increasing attention in Romania—Cluj Parking, Suceava Parking, and Parking București, to name a few. For example, Cluj Parking is designed to work with barrier parking lots only. The access controller can count the number of times a barrier rises at the entrance and exit. In theory, a simple subtraction can give the number of occupied spots. In practice, this method is error-prone, and it does not offer the possibility to make statistics at a spot level. Suceava Parking does not provide availability data—its main function is parking payment. Parking București has “live” availability that a person manually updates. 

A common feature of all these solutions is the ability to start guided navigation to the selected parking lot. While these solutions are a step forward, they are too many steps away from the concept of smart parking, let alone as a Parking Guidance and Information System (PGIS) [4] part of an Intelligent Transport System (ITS) [4] in an urban environment. 

Based on our observations, for smart parking solutions to affect the quality of life in an urban area, over 30% of the parking places need to be integrated with a platform that offers the public at large the following features:

- Near real-time (under 2 s delay) data of parking spots availability—anyone should be able to easily see if parking spots are free in an area.
- Location-based services—showing services based on someone’s location.
- Reservation and payment—using a mobile device, one should be able to reserve a parking place and pay for it.
- Statistics and predictions—useful for end-users and decision-makers.
- Scalable and easily deployable—the platform must work the same with 10,000 parking spots as it would with 100,000; also, it should be easy to add new parking lots in a timely fashion.

### 1.2. Improvements to the Current Model, Although a Traditional Implementation

In 2017, VA Solutions Labs launched the Parking Spotter project [6], a smart parking solution [4]. The project’s initial focus was to use the security cameras as a video feed source for a computer vision algorithm and detect vacant spots. Initially, the solutions integrated only with the security solution to trigger an alarm in case specific parking areas were occupied. 

**Figure 3:**
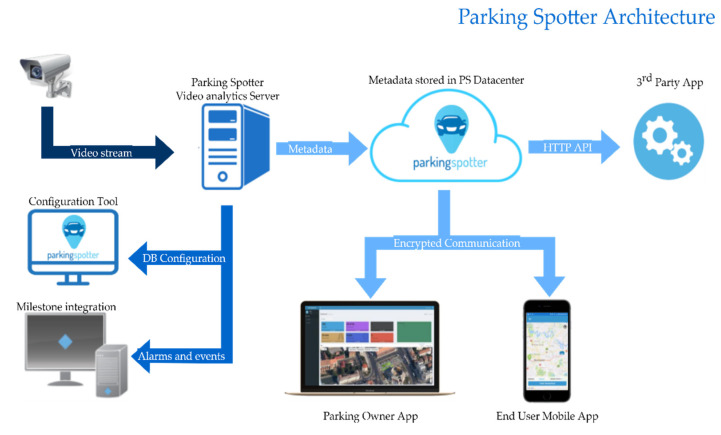
Parking spotter architecture.

After noticing the possible information flows, we designed a modular smart parking platform that can connect sensors, mobile devices, drivers, parking owners, and municipalities. The final architecture of the Parking Spotter is presented in Figure 3.

From an Information Collection [4] standpoint, the platform is device agnostic and can easily integrate with any stationary sensor. As in Figure 3, the Data Analytics Server acts as an on-site integration that translates data to a format acceptable by our API (Application Programming Interface). The parking’s initial configuration is the process that maps physical devices to LPS (Logical Parking Spots): for example, mapping a magnetometer sensor’s hardware id to an LPS unique identifier. LPS’s basic attributes are GPS location and status.

“System deployment deals with the software system exploitation and the statistical analysis of the collected data. Software generally involves E-Parking, reservation, guidance, and monitoring information for administration and users. With the collected data, an analysis is often performed by data scientists to study drivers’ behaviors to improve the system performance.”

[4]

As shown in Figure 3, Parking Spotter checks all the main points mentioned in the quotation above. A driver can use the mobile app to see nearby parking lots and the available spots. Then, they can reserve a spot and start a navigation app to the GPS coordinates. If someone drives to a place reserved by a mobile user and does not use the mobile app, the system notifies the patron that they are redirected to a nearby parking spot. The parking owner has reports and real-time data at their disposal in a dashboard web application. 

Presently, Parking Spotter [3] has had only implementations of private parking lots but has been publicly available. A detailed analysis of the relationship between information and social features is not commercially feasible without a partnership with a municipality. Although, the notion of Service Dissemination [4] makes sense for a large economic agent with multiple parking lots spread across a larger surface area, in this case, an important factor is a narrow timeframe for project implementation.

“Machine vision is another technology which uses visual camera to acquire real time parking occupancy information on open parking lots due to its minimal expenditure. The usage of some visual cameras is dependent on regulations supported by the country, which needs to be considered prior. However, there is no single ideal technology suitable for parking occupancy detection. Based on the type of parking lot and size, different combinations of smart parking technologies and sensors can be used for efficient and financially viable parking occupancy detection.”

[4]

From the standpoint of usability and incentive structure, we noticed the following possible risks and problems:

- No matter how good the hardware or software on-site implementation is, the-end user will not use it if the mobile app is bad.
- With a very good mobile app and service, the end-user will not use it if there are only a handful of parking lots are available on the platform.
- A badly administered implementation will overshadow the best possible solution.
- Without the possibility to make price discovery, investors lack interest in developing new smart parking lots.
- Without macro statistics and large-scale user adoption, municipalities are reticent of investing in a project of which the success rate cannot be measured.

### 1.3. Challenges

Technical issues have been thoroughly studied in the literature, and, in many instances, solutions have been proposed for smart parking and live availability. We found no mention of smart parking networks that will work for greater geographical areas, similar to Parking Guidance and Information System (PGIS) [4] as part of an Intelligent Transport System (ITS) [4] in an urban environment. A network is greater than the sum of its parts.

Current centralized services inhibit collaboration and interoperability at the expense of user adoption. Parking providers compete to capture the end-user in their respective ecosystems but fail to understand that a collaborative network can only resolve the mobility problem. Service discovery is not facilitated. A driver interested in parking in a particular area must check with one or more mobile applications to discover the location of parking lots. Generic navigation applications offer geolocated information but lack live availability, context, and metadata (how many total spots, electric charging availability, etc.).

Common actions (service discovery, queries based on geolocation, live availability, and parking spot reservations) should be available on all user interfaces. The user experience can be different, but the interaction with the system can be abstract. Current smart parking implementations do not permit access to third-party parking services. The end-user is forced to have multiple accounts and, thus, leave his personal information with various parking providers. The simple task of finding available parking should not require this. The operators justify this with the need to protect against bad actors.

The business model for parking providers is inefficient, as IT maintenance and software development for user interfaces require resources that could be better spent on their main focus—parking. Another important challenge is the system’s resilience, as most have a single point of failure. As Raymond Hettinger famously affirms: “There has to be a better way!” [7].

### 1.4. Our Proposal

We propose a trustless federated system built upon Zero Knowledge Proofs [8], Graphs (inspired by Raymond Hettinger [7]), and IPFS. We normalized the underlining data model, created an abstraction for the user to platform interaction, and associated the end-user with an identity based on Zero Knowledge cryptography. The main goal is to lower the development effort for user interfaces (mobile apps, car apps, and web apps) and create similar a user experience across different applications. Nonetheless, by building upon existing technology, we highlight an effective way to establish a verifiable trust relationship between the parking providers—the entities that expose the actual resources to the network.

The structure of our paper is as follows. We describe, in detail, our proposal in Section 2, Methodology, which is structured in subsections as follows: Section 2.1, Federated Smart Parking Network, briefly describes the overall architecture; Section 2.2, Zero Knowledge Federated User Account, presents the mechanism that allows for an anonymized identity to interact with previously unknown parking providers; Section 2.3, Static data versus Live data, describes a resilient mechanism of distributing data that seldom changes (parking description, location, and metadata) with the help of IPFS; and, in Section 2.4, Parking Application Models and Protocol—PAMP, the kernel for user interface interaction is detailed. In the last subsection, Section 2.5, Recipe for interaction between Parking Providers—RiPP, we present the mechanism that encourages collaboration between parking providers without sacrificing their autonomy. Section 3, Discussion, synthesizes the major features of our approach and presents a meaningful comparison with another two methods from the literature. Further research elements are also included here. Finally, Section 4, Conclusions, summarizes the paper.

## 2. Methodology

This section describes the structure of the proposed Federated Smart Parking solutions and explains in detail its main components, their operations, and interrelations.

### 2.1. Federated Smart Parking Network

**Figure 4:**
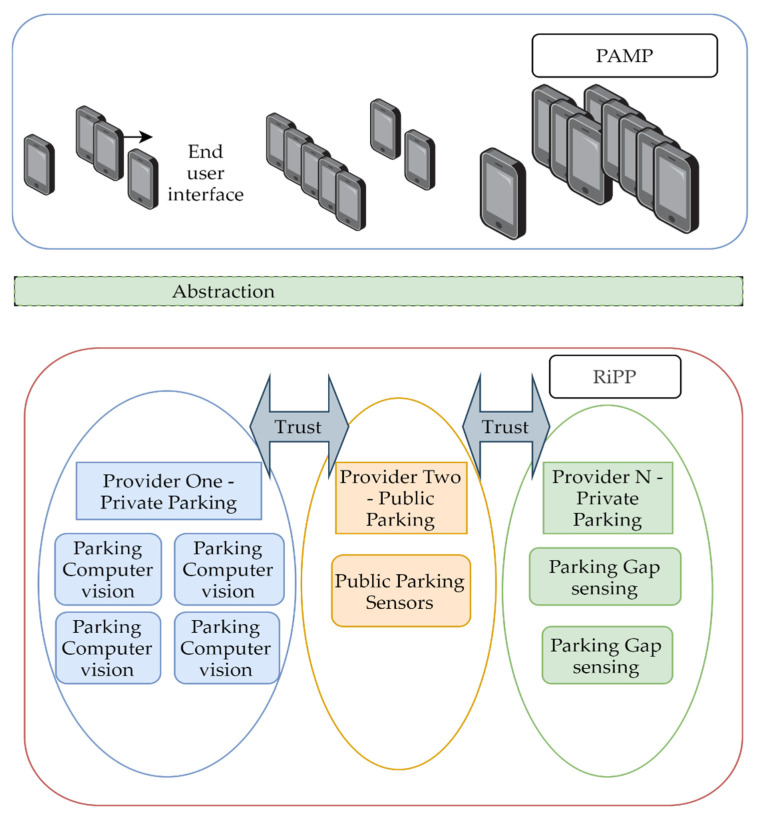
Federated smart parking model.

We propose a trustless decentralized system based on a federated architecture. A trustless system, in this case, is an open network that allows verifiable interactions on predefined rules. Trust is not eliminated but instead is distributed between participants as it does not require a central authority to function. A service provider is considered the source of truth for its own parking lots. Any node of the federation can offer information about the networks resources and redirect the user to the authority for the required resources. Interactions between participants are verified by cryptographic algorithms. The architecture consists of two main components (Figure 4):

- Parking Application Models and Protocol (PAMP) will expose information to the public.
- Recipe for interaction between Parking Providers (RiPP).

Each bubble represents an abstraction layer between users, information, and technology. The blue bubble in Figure 4 contains all the mobile applications implementation that use the PAMP to interact with data from parking providers. Using a common data model and protocol, developers can implement a user interface not depending on an operating system or programming language. Every parking provider (PP) in the system must offer:

- A querying mechanism for parking lots in a geographical area.
- Live availability of the spots of a certain parking lot.
- Reservation of parking spots from mobile apps.
- Mechanisms to publish events between trusted parties.
- A public key.
- A token for each client for authorization.

### 2.2. Zero Knowledge Federated User Account

All of the above must be done without compromising the end-user security and protection of private data (phone number, email, etc.). A relationship between parking providers can be established using a public–private key pair; however, for the sake of performance and to inhibit user tracking between PP, we will employ a Zero Knowledge (ZK) Proof mechanism [8].

Assume a certain client, client C1, is registered with PP P1, which knows the client’s personal data. C1 authenticates and receives a token T1 from P1. Every request to P1 contains the authorization token. C1 does a spatial query for the city he is visiting. Parking provider P1 does not own any parking lots in that city, but PP P2 that P1 has a trust relationship does and responds to the C1 to inform him on parking lots under P2 administration. 

**Figure 5:**
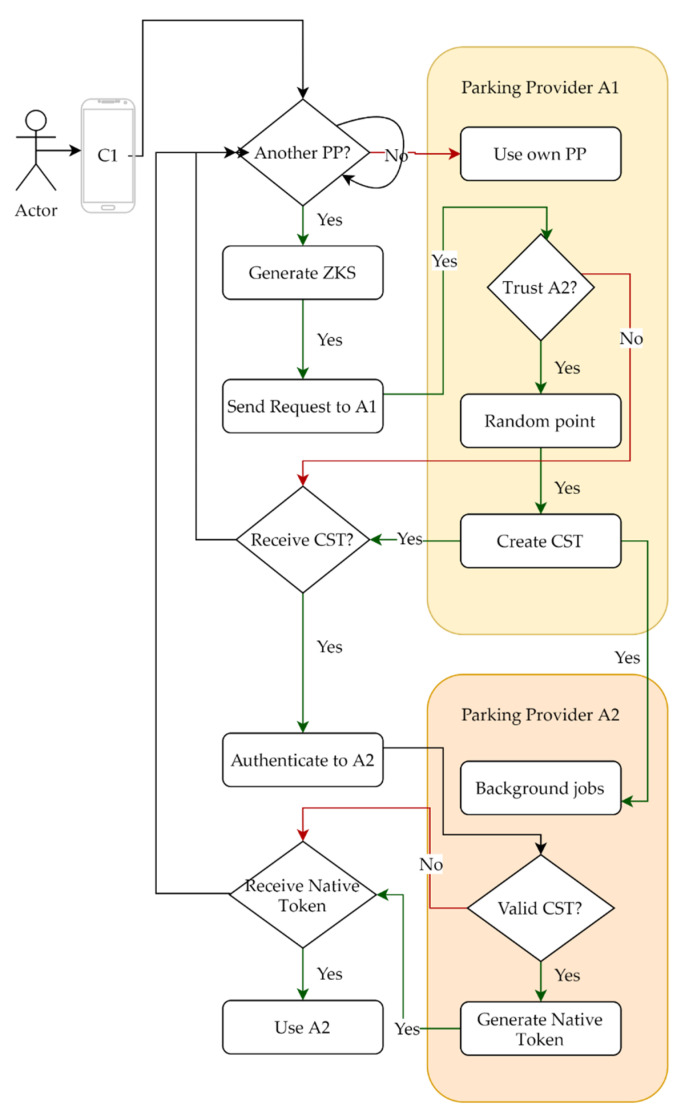
Mobile app requests PP one to envelop ZK signature and notify PP two.

A diagram of the interactions between the Mobile App and PP, is presented in Figure 5. The mobile app creates a virtual identity using a Zero Knowledge signature (ZKS) derived from the pair (C1 secret and P2 public key). ClientC1 asks his parking provider to sign ZKS1 and to notify that C1 will use its services. The parking provider P1 envelopes CST1, a random point on the elliptic curve of ZKS1 and other meaningful data (expiration date, audience, etc.) in a JSON Web Token (JWT) [9], signed with its private key—we will call this a client signature token (CST). In the background, there are a lot of actions taking place, but as soon as C1 receives CST1, it can compute a ZK Proof and authenticate to P2 only once.

An extraordinarily strong point for this approach is that the user cannot be tracked between parking providers. User C1 can interact, using a different virtual identity, with P1, P2, P3, and P4. Only P1 (to which the user consented to personal data usage), the provider that guarantees it access to the network, is aware of the client’s interaction.

#### 2.2.1. Authentication to Trusted 3rd Party

The user’s app sends CST1 and ZK Proof to the authentication endpoint of P2, as seen in the second part of Figure 5. The PP checks CST1 for the signature and validity, then continues to validate the Zero Knowledge Proof, and in the end returns a JSON that includes:

- a new random point on the elliptic curve of CST1, used as refresh token and
- a native JWT that C1 will add with every authorization request.

Essentially, the mobile app C1 (prover) claims that it knows *x* such that *B* = *x* · *G* (as explained in step 1 below), and it wishes to prove this fact to Verifier (PP P2) without revealing *x* [8]. Even though PP P1 acts as a guarantor for C1, it cannot impersonate C1. The process, step by step, is:

1. The mobile app C1 will pick an elliptic curve model (*E* over field $\;F_{n}$ characterized by a generator field $G\;\in E/F_{n}$ and $P\;\in E/F_{n}$ ) and use a ‘secret identifier’, x, to create a ZKS based on Schnorr’s Protocol [2].
2. C1 will ask P1 to sign data describing *E* (*G*, *P*) and B. P1 picks random point $r\;\in F_{n}$ necessary to compute *A = r · G*, then signs and returns a JWT if there is trust, and P2 accepts clients.
3. C1 uses random point $r\;\in F_{n}$ to compute *A = r · G*, then *C = HASH(x · P, r · P, r · G)* and finally *s = r + c · x(mod n)* and sends to P2 message “*s||x ∙ P||r ∙ P||r ∙ G*”, a ZK Proof, with the JWT from step 2 to authenticate.
4. Verifier P2 checks to see if the JWT is valid.
5. Verifier P2 computes *C = HASH(x · P, r · P, r · G)* and checks that
  s·G=A+c·Bs·P=r·P+c·xP
6. P2 saves the virtual identity and generates a new random number *r*’ to be used for a refresh.
7. P2 creates a native JWT, signs it, and returns it to C1.

From this point onwards, the mobile app can query live data methods and create a spot reservation.

#### 2.2.2. Native Token Refresh

By providing the native token and a new ZK Proof based on the random number from the authentication step, C1 can continue to use P2 resources. Like authentication, the response to this request will contain:

- a new arbitrary point on the elliptic curve, used as refresh token and
- P2’s native JWT to be used with every authorization request.

#### 2.2.3. Banning a Virtual Identity

Abusive behavior, such as repetitive cancelation of spots reservations or missing reservation deadlines, will lead to a permanent block of a virtual identity. Mobile apps can easily request another virtual identity. Suppose the trusted party does not carefully manage the creation of multiple identities for the same client. In that case, it could lead to a temporary block of all clients and eventually to a trust breakup. 

### 2.3. Static Data versus Live Data

There are two types of data: static and live. Static data could be considered any information that is not expected to change in the near future:

- Parking lot name and location.
- Parking spot location.
- Authoritative information about the parking lot: who manages it, the URL of the PP endpoints, the public key, etc.
- The history of the relationships between parties: who trusted who, have they shared information, etc.

This information could be made easily available over HTTP. Several problems could appear in this instance. In most cases, HTTP has a single point of failure and was designed by the principles of addressing content by location (location based or location addressed). When a data system relies on location, it is about querying a service by its host name using a DNS server. This tracks a host by a logical addressing scheme (e.g., IP address) mapped to a user-friendly name. If the host changes its name or address, it must also be modified in the name service table.

If DNS resolver fails, the service fails. If the IP address changes unexpectedly, the service will become unreliable, plus the logical addressing scheme does not allow for data to be moved closer to the client. By cloud, providers have built Content Delivery Networks, but these kinds of solutions raise the system’s complexity and rely on third parties. To make things worse, a bad actor could take advantage of the mutability of the data in a location addressed system and change the information on the server without hinting the client.

Static data, available at an Interplanetary level, require another Storage Addressing Scheme—these data require an InterPlanetary File System (IPFS). The main characteristic that sets IPFS apart from typical cloud-based storage systems is that it is content-based (content addressed) and not location-based (location addressed).

In the case of speed or reliability, IPFS (http://ipfs.io, accessed on 24 June 2021) can perform better than traditional HTTP, as it can provide the file from various servers (e.g., a peer or node on the IPFS network) that are near to the user. Rather than rely on a server location to obtain a file, a user can simply search for a data object without a search engine referencing the location, i.e., the server name or address. Instead, they will reference it by the file’s hash, and it will be available from the nearest available nodes on the network.

“IPFS is a distributed file system which synthesizes successful ideas from previous peer-to-peer systems, including DHTs, BitTorrent, Git, and SFS. The contribution of IPFS is simplifying, evolving, and connecting proven techniques into a single cohesive system, greater than the sum of its parts. IPFS presents a new platform for writing and deploying applications and a new system for distributing and versioning large data. IPFS could even evolve the web itself.”

[10]

The main IPFS functionalities are:

- Identities—every node is identified by a NodeId, a hash digest value stored in a multihash [11] format, created with a S/Kademlia’static crypto puzzle [12].
- Network—manages peer-to-peer connections, using various configurable network protocols.
- Routing—responds to local and remote queries, based on Distributed Hash Tables (DHT) [13], keeping track of peers and objects.
- Exchange—uses BitSwap [10] to keep a ledger with the exchange of data blocks between peers to prevent abuse of bandwidth.
- Objects—Arbitrary data are represented using Merkle DAGs [14] as immutable objects with links to tamper-resistant data blocks.
- Files—like Git, it offers the possibility to have a versioned file system.
- Naming—Self-Certified Names [10] have a mutable link to permanent objects. Based on a public–private key pair, a self-certified name can be constructed in a cryptographically assigned global namespace and, by using the Routing system, with a mutable state distribution, establish a link between a mutable path and an immutable object.

In his article, Konrad Hinsen clearly presented the concept of naming data [15], be it mutable or immutable. For example, we have a list of numbers [1,2,3]. An IPFS CID is essentially a hash; however, it contains additional information. The initial ‘b’ indicates that the CID has been encoded as a number in base 32. Base 32 uses only the 32 characters abcdefghijklmnopqrstuvwxyz234567/and ignores the case. This is done to assure it will travel unchanged over Internet transmission channels that might strip spaces and control characters or convert everything to upper case. Decoding the base 32 string yields the real CID, a sequence of 36 bytes as a hexadecimal notation. The first byte is a version number. Future extensions to the CID format will not invalidate existing CIDs.

Our proposed architecture attempts to fully capitalize on the benefits of the Inter-Planetary Name System (IPNS). Data will be published between parties over IPFS, as each PP will have a SCN (Self Certified Name). At the moment, mobile devices lack a stable version of IPFS, but viable solutions seem possible shortly. Until then, information transfers between mobile devices and PP will be done over HTTP. We can assume that PP can publish static data as secure and verifiable. If all the participants use IPFS, then the data transferred between participants will be lowered drastically. 

When a user queries the PP about parking lots in a particular area, he receives the SCN of each parking lot and the authoritative PP. A node checks the link between a SCN and a CID (content identifies), caching the blocks in the process. Now, the user can view static data about the parking (name, announcements, business hours, number of spots, entrance coordinates, etc.) multiple times without generating multiple requests. The same benefits apply for data transfers between PP—not every user query for static data will lead to an exchange of data. 

Live data are time-dependent information: if a parking place is full in the moment of querying, a user that competes with others for a parking place may make a promise to occupy a spot at a future time could be canceled.

In the case of public parking with free access, the live availability map of parking places would tell the end user how many are free and give the possibility to make a reservation for a spot S1. This will not guarantee that, on arrival, the beneficiary will occupy S1—it might be rerouted to S2. Mechanisms to notify end-users of events that affect them, are a requirement. IPFS has a pub-sub feature that can be used, but it is impractical for mobile devices. In the case of mobile devices, for live data, we must use HTTP, WebSockets, or push notifications. 

Even if IPFS is used only for static data, the parking services could operate in an offline mode, as the data about PP P1 will be available at trusted PP P2. 

### 2.4. Parking Application Models and Protocol—PAMP

Next, we will describe the data model and the sets of conventions governing the interaction with the system’s methods. 

#### 2.4.1. Data Model

We list the main attributes and data types.

Parking Lot Data Model:

- ParkingLotName: 128 characters UTF-8, the parking lot name.
- ParkingLotDescription: 512 characters UTF-8, the parking lot description.
- ParkingLotGuid: Integer 64 bits, a very large integer that identifies the parking lot almost uniquely in a large geographical area.
- ParkingLotType: Unsigned Integer 32 bits, an array of attributes will be binary encoded (for example, if the parking is private or not, if it is with pay, if it has charging stations, if it has spots for people with special needs, etc.).
- ParkingLotX: Decimal float with six decimal places to describe the latitude of the entrance.
- ParkingLotY: Decimal float with six decimal places to describe the longitude of the entrance.
- ParkingLotCentroidX: Decimal float with six decimal places to describe the latitude of the lot’s center.
- ParkingLotCentroidY: Decimal float with six decimal places to describe the longitude of the lot’s center.
- ParkingLotSpotsNo: Unsigned Integer 32 bits with the total number of spots.
- ParkingLotActiveAt: Date time string of the last activity check.
- ParkingLotStatus: Unsigned Integer 32 bits.

ParkingSpotData Model:

- ParkingSpotName: 128 characters UTF-8, the parking spot name or description.
- ParkingSpotGuid: Integer 64 bits, a very long integer that identifies the parking spot almost uniquely in a large geographical area.
- ParkingSpotX: Decimal float with six decimal places to describe the latitude of the spot.
- ParkingSpotY: Decimal float with six decimal places to describe the longitude of the spot.
- ParkingSpotType: Unsigned Integer 32 bits, an array of attributes will be binary encoded.
- ParkingSpotArea: 128 characters UTF-8, the parking spot’s sector or grouping description.
- ParkingSpotStatus: Unsigned Integer 32 bits.

#### 2.4.2. List of Methods to Request Data

All methods accessed by clients on any of the PP require a token for authorization. 

The full path URL is comprised of a domain and a method path. The responses have the content-type ‘application/json’ and encoded as utf-8. Static data can be accessed by IPNS using /ipns/{ParkingSCN}/{method path}. The URL format is used for backward compatibility. All static methods can also be queried over HTTP. There are restrictions on the technology used to build the user interface.

The following list contains the name of methods to request live data and their path:

1. parkingNearby (/parking/nearby)—a spatial query that returns the parking lots that are within the distance D from a point P(X,Y) characterized by a decimal latitude and longitude values; parking places from trusted PPs are also returned.
2. parkingAllSpots (/parking/{ ParkingLotGuid }/spot/all)—returns all the available free spots from a parking lot.
3. parkingSpotPending (/parking/{ ParkingLotGuid }/spot/{ ParkingSpotGuid }/pending—a client notifies the PP of the intention to reserve a spot—a probability between 0 and 1 is returned and preconditions (you need to pay in advance a small fee, etc.).
4. parkingSpotReserve (/parking/{ ParkingLotGuid }/spot/{ ParkingSpotGuid }/reserve—a trustworthy client will receive a confirmation.
5. parkingSpotUpdate (/parking/{ ParkingLotGuid }/spot/{ ParkingSpotGuid }/update—method used by sensors or integrations to trigger a spots status change—from free to occupied and vice versa.
6. checkProof (/interaction/proof)—a client authenticates to a PP using a ZK proof.
7. requestSign (/interaction/sponsor)—a client requests its authoritative PP to sign the ZKS.

Using the live methods enumerated above, an application implementation can request a list of parking lots near a GPS location and check if there are free spots. 

Static data are available over IPFS at:

1. wellKnown (/authority/wellknown)—returns the public key of the current PP and other data that describes the authority.
2. wellKnownPP (/authority/wellknown/pp)—an array of trusted PP with their public keys, domains, and other data describing the PP capabilities.
3. parkings (/authority/parkings)—returns data about parking places under the administration of the current PP, such as parking SCN, description, coordinates, etc.
4. requestTrust (/interaction/trust)—a mechanism to start the establishment of trust between parties and generate a two-way handshake.
5. requestChallenge (/interaction/challenge)—trust is established and published for other parties to inspect.
6. requestNotify (/interaction/notify)—a PP notifies another of a client that requested access to its resources or if an identity is revoked.

### 2.5. Recipe for Interaction between Parking Providers—RiPP

Interaction between parking providers is governed by simple rules described in RiPP and secured by cryptographic means. No central entity validates a trust chain or restricts access to the network. Each entity establishes trust at a peer-to-peer level and chooses to extend trust to “friends of friends” by its own rules. A trustless network is created, governed by a graph of trust contracts cryptographically signed.

#### 2.5.1. Establishing Trust between Providers, but Not between Clients of a Provider and Others

As per definition, interaction is “*a situation where two or more people or things communicate with each other or react to each another*” [16]. We assume the parties involved as the Parking Providers. Each PP has authority over a physical space (parking lots) and cyberspace (domain, servers, public–private key pairs, IPFS node, and identity). We will focus on the cyberspace component and provide a recipe for exchanging and verifying proofs of authority 

Each PP must prove it has authority over a domain. The mail DomainKeys Identified Email (DKIM) mechanism could be a good source of inspiration. It provides a way to certify that an organization delivering an email has the right to do so.

**Figure 6:**
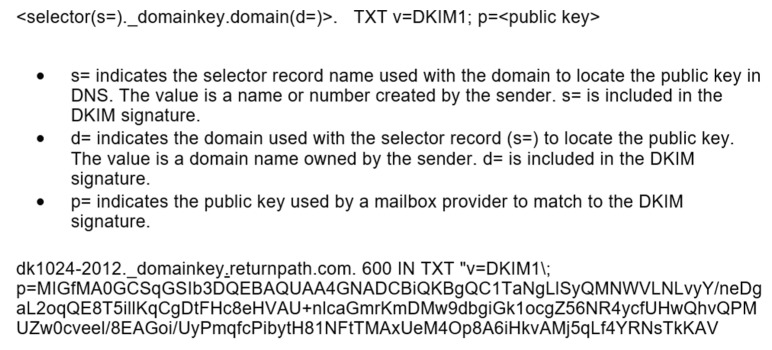
DKIM template, source webpage [17].

In Figure 6, we have presented the structure of a DNS record utilized for DKIM verification. A valid PP must have a DNS TXT record similar to a DKIM one, with the mention that “v = RiPP1” should replace “v = DKIM1”. The public key, version, and other data should also be available at the well-known method. 

It must be easy to verify and obtain the public key of an operator from different sources. The PP public key is not the same as the IPFS public key. IPFS can look up for IPNS in DNS TXT records—DNSlink [18] is recommended. A PP must have a DNS TXT record as follows:

*“_dnslink.parkingspotter.com. 34 IN TXT dnslink = /ipfs/QmVMxjouRQCA2QykL5Rc77DvjfaX6m8NL6RyHXRTaZ9iya”*

All described conditions must be met for a PP to be considered valid and for a trust relation to be established. Either of the involved parts can initiate a trust relationship. At first, there might be a signed agreement between parties. In this scenario, Parking Provider P1 initializes the process of establishing the relationship between parties.

Steps to follow for establishing trust:

1. PP P1 verifies that PP P2 checks the DNS and IPFS prerequisites.
2. A human operator from PP P1 uses the **requestTrust** method with PP P2. The request includes information about the requester, a random number of 128 bits (RND1) encrypted with the public key of PP P2, a ZK signature, and other useful data. The requests data are signed using a PP P1 private key. Optionally, the public key can be included in the request.
3. PP P2 checks the validity of the signature and verifies that PP P1 checks all the prerequisites. It verifies that the same public key can be found in DNS records, on IPFS, and in the request. It decrypts the random number RND1 using the private key. If prerequisites fail or the number cannot be decrypted, the process stops.
4. A human operator from PP P2 triggers a **requestChallenge** for PP P1 that includes information about the requester, a random number of 128 bits (RND2) encrypted with the public key of PP P1, a ZK signature, an AES256 encrypted data (random generated data used as a secret challenge SeCh) with the encryption key being composed of the two random integers RND1 || RND2.
5. In an automated manner, PP P1 will decrypt the SeCh and then confirm by triggering a **requestChallenge** for PP P2, which includes information about the requester, the inverse of RND1 encrypted with the public key of PP P1, a ZK signature, and AES256 encrypted data reversed SeCh with the encryption key composed of the two random integers reverse (RND1 || RND2).
6. At the last step, PP P2 will call **requestTrust** sending only the current UTC datetime as ISO string to signal, encrypted with RND1 || RND2.

**Figure 7:**
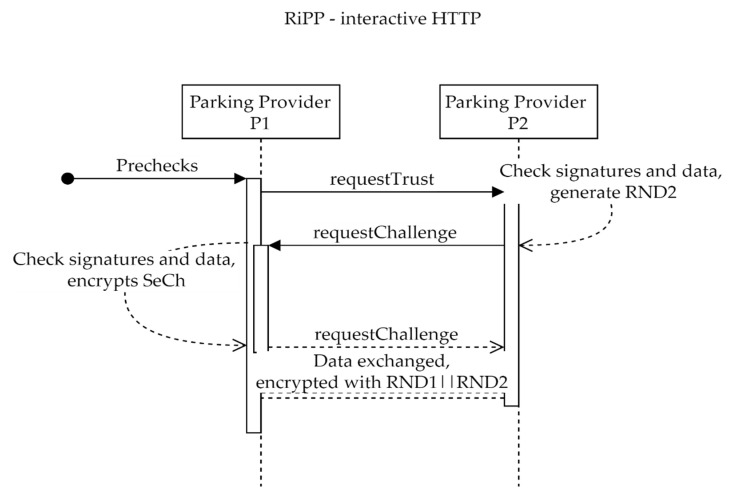
RiPP interactive.

The described recipe can work in interactive (or live) mode using HTTP (see Figure 7) or in non-interactive mode using IPFS. 

The interactive mode uses the standard REST recipe—one server calls the methods of another following the recipe. Any data that need to be encrypted will use RND1||RND2 as a secret key. A key derivation function may be employed at a later stage.

**Figure 8:**
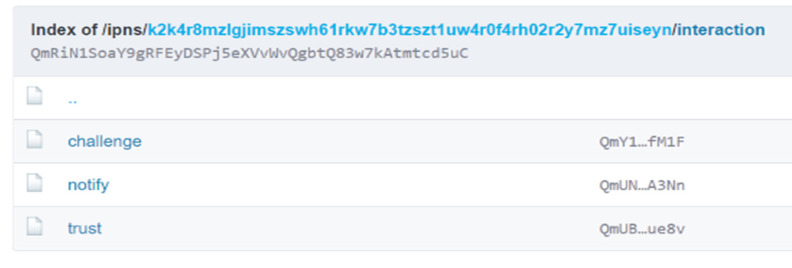
IPNS example.

The same recipe can be applied in a non-interactive way using IPFS. One can treat the two methods with a common base as a folder or a hierarchical folder structure. On the first level, we have an IPNS public key that points to a CID, such as “k2k4r8mzlgjimszswh61rkw7b3tzszt1uw4r0f4rh02r2y7mz7uiseyn”, as seen in Figure 8.

**Figure 9:**
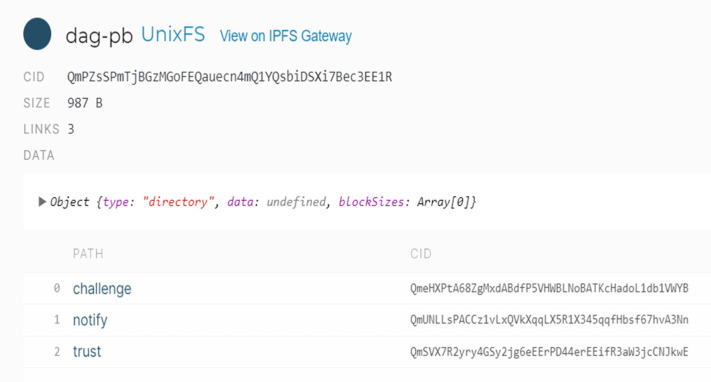
Interaction methods as unixfs.

The hierarchy continues:

∙ Interaction (see Figure 9)
  ○ trust
    ▪ files for each request or response
  ○ challenge
    ▪ files for each request or response
  ○ other folders
    ▪ some other folder
      ∙ files
  ∙ authority
    ○ well-known
      ▪ pp
        ∙ files or links to trusted parking providers
      ▪ files for the current parking provider
      ▪ parking
        ∙ files or links to parking lots of current parking provider

Parking providers will need to follow roughly the same six steps from the interactive mode:

1. PP P1 verifies that PP P2 checks the DNS and IPFS prerequisites.
2. A human operator from PP P1 uses the **requestTrust** method with PP P2, but instead of calling a HTTP method with the data, it will save the data in **/ipns/{ParkingSCN}/interaction/trust /{naming convention .request}**. The request file includes information about the requester, a random number of 128 bits (RND1) encrypted with the public key of PP P2, a ZK signature, and other useful data. The request data are signed using a PP P1 private key. Optional, the public key can be included in the request. The requester notifies the other party over other communication (mail, phone) channel that a request has been made, indicating the SCN.
3. A human operator from PP P2 activates a background job for P1’s SCN, verifies that PP P1 checks all the prerequisites. When it finds the request file, it checks the validity of the signature and it verifies that the same public key can be found in DNS records, on IPFS, and in the request. It decrypts the random number RND1 using the private key. If prerequisites fail or the number cannot be decrypted, the process stops.
4. PP P2 triggers a **requestChallenge** for PP P1, that includes information about the requester, a random number of 128 bits (RND2) encrypted with the public key of PP P1, a ZK signature, an AES256 encrypted data (random generated data used as a secret challenge SeCh) with the encryption key being composed of the two random integers RND1 || RND2. Data are saved in a request file at **/ipns/{P2′s SCN}/interaction/challenge/{naming convention request}**.
5. Automatically, PP P1 will check at regular intervals if P2 has written a response on IPFS. As soon as it identifies the file, it decrypts the SeCh, then confirms by triggering a **requestChallenge** for PP P2 that includes information about the requester, the inverse of RND1 encrypted with the public key of PP P1, a ZK signature, and AES256 encrypted data reversed SeCh with the encryption key being composed of two random integers reverse (RND1 || RND2). Data are saved in a respose file at **/ipns/{P1’s SCN}/interaction/challenge/{naming convention response}**.
6. PP P2 detects the response file from step 5, checks if the data are valid, in which case it seals the agreement with a response file that contains the current encrypted UTC datetime, at **/ipns/{P2’s SCN}/interaction/trust /{naming convention response}.**

Using the non-interactive mode, the same data are passed around and validated in the same way—the only difference is that data are transmitted using IPFS. The naming convention addresses the method of naming a file to associate it easily with one entity or another. A basic naming convention would be: YYYYMMDD||SEPARATOR ||SCN–example20210316__k2k4r8mzlgjimszswh61rkw7b3tzszt1uw4r0f4rh02r2y7mz7uiseyn. 

At this stage, the two parties agree to trust each other, exchange encrypted data using a secret key RND1||RND2, and sign data using their unqualified public–private key pairs. 

#### 2.5.2. A Network with Different Levels of Trust

**Figure 10:**
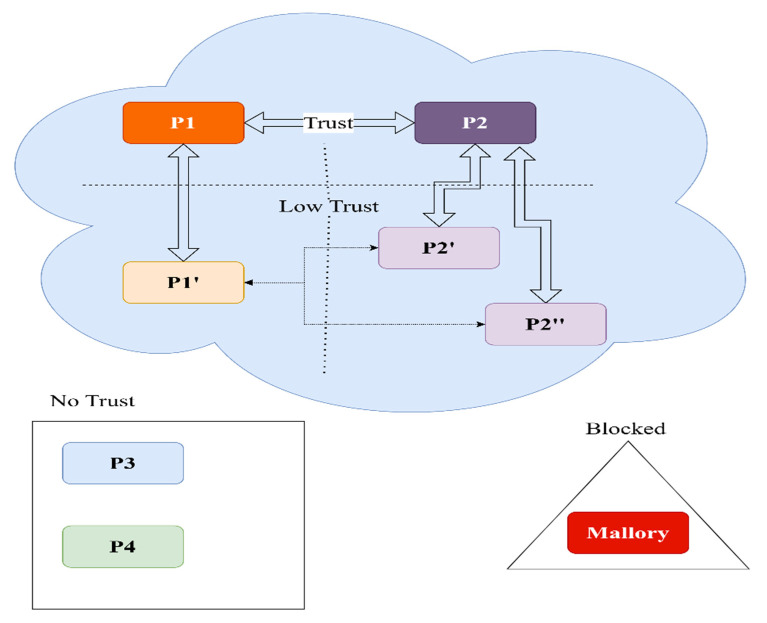
Trust relationships.

There are several levels of trust; with increased levels, more interactions are possible (see Figure 10).

We propose the following list, starting from the lowest level:

1. Blocked—this PP is considered an abuser and is blocked.
2. No Trust—this is the default.
3. Low Trust—the PP is in the well-known providers list of a trusted entity; no agreement has been signed between the parties, but the prerequisites for establishing trust are fulfilled.
4. Trust—an agreement was signed between parties that were in contact and have covered the six steps described in the aforementioned recipe.

**Figure 11:**
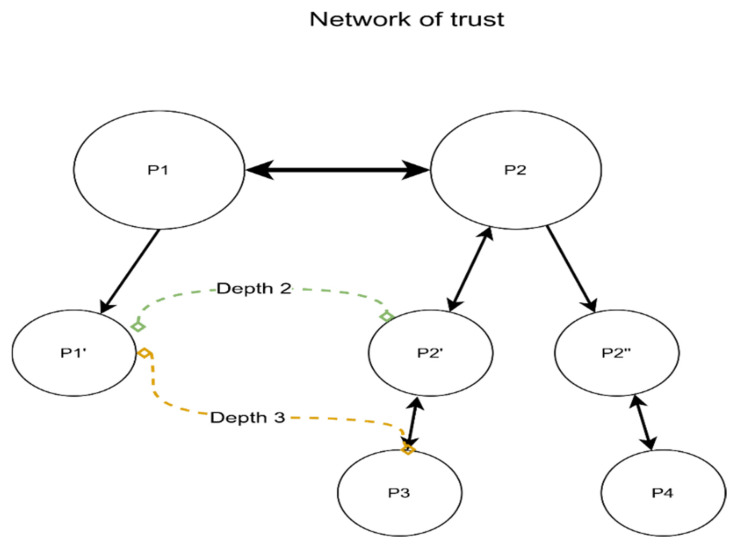
Network of trust, a directed graph.

The trust relationships could be treated as a directed graph (Figure 11). If the edge is bidirectional, both parties have registered users that will use each other’s parking lots. Otherwise, the edge will be from the PP that manages users to the PP that manages only the physical parking location. The main beneficiary of the trust network is the user that wants to consume a resource—the parking spot.

Depending on the agreement, P1 and P2 could choose to trust each other’s partners. As described in Figure 11, P1 could consider low trust P2′ and P2′. In the same manner, P2 will consider low trust P1′. We consider this to be a trust depth of one. Let us assume P1′ is the junior partner (does not manage users) of P1 and will accept a trust depth of two. 

A direct trust relation is considered a level zero or zero depth—example P1 → P1′. Users from P1 can use P1′ resources, but P1 will not accept users from P1′. The junior partner relies on the senior partner to send customers. We can observe, in the graph represented in Figure 11, that P2 → P1 → P1′ is a valid path, as is P2′ → P2 → P1 → P1′. The latter has a trust depth of two and gives users from P2′ access to P1′ resources. In the case of a depth of three, there is a valid path from P3 to P1′. None from P4 can use the spots, as the path P4 -> P2′’ → P2 → P1 → P1′ is invalid. The directed graph is easy to construct and does not change often. 

In practical terms, as a possible case, think of a PP from Tokyo (assume P2) that created a mobile app for its users—the app is in Japanese. Bucharest is a popular destination for its users, so a trustworthy PP (assume P1) is found, an agreement is signed, and RiPP is implemented with the trust depth of two. Now, all the users of P2, P2′, and P3 can use their own apps to consume resources from P1 and P1′. The trusted parking providers from Bucharest receive more customers, the end-users are happy to find parking spots in the same app they used in Tokyo, and the city will have fewer drivers cruising around for a free spot.

#### 2.5.3. Possible Problems and Mitigations 

Mechanisms to inhibit abuse must be put in place. A malicious actor could hack a trusted party and spawn free spot reservation requests to exhaust the target’s resources. We assume that a mobile app client queries free spots, marks one for reservation, drives to the parking lot, and parks on the reserved spot.

In the case of an attack, real clients will see that the PP does not return any available parking places and will assume everything is occupied. This can have terrible consequences from a commercial or branding standpoint. Allowing one ongoing reservation with a PP per virtual identity will limit the vector of attack. Repeated attempts to create a reservation and then cancel it are easy to identify and block. If the PP is compromised, unlimited virtual identities can be generated, each viable for a reservation. 

We take inspiration from the Bitswap strategy [10] and attempt to keep a parking provider Ledger. When a PP’s clients generate bad reservation requests, a temporary block will be triggered.

A spot reservation can have the following statuses:

- PENDING—a client is on route to the parking lot.
- FULFILLED—end-user is currently occupying a parking spot.
- CANCELED—a client has actively canceled a reservation.
- UNFULFILLED—a timeout occurred, and the client did not make it to the destination.

Consider *b* symbolizing the sum of reservation requests CANCELED or UNFULFILLED, while *g* stands for the sum of PENDING or FULFILLED requests.

$$k=\frac{b}{g+1}$$

We calculate probability *P*, which determines if a client from a particular PP can reserve a parking spot, as follows:

$$P=1-\frac{1}{1+e^{6-3k}}$$

**Figure 12:**
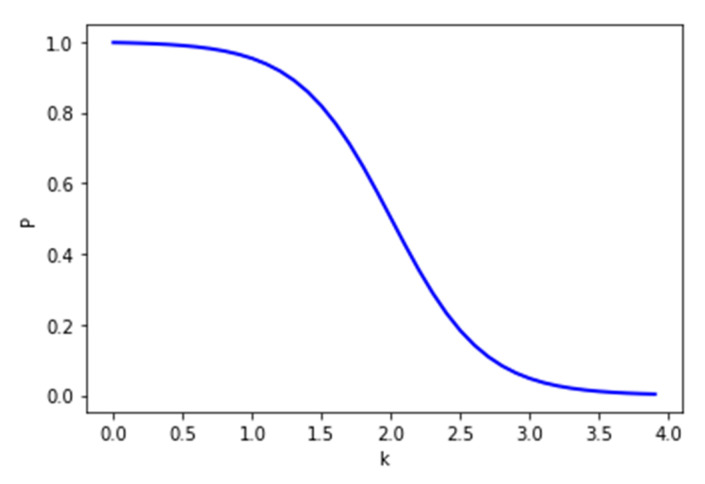
Probability of reservation to be accepted.

When the kill ratio (*k*) exceeds value 2 (Figure 12), the probability *P* drops significantly. The ratio can be reset after a cooldown period.

Depending on *P*, from a reservation ledger standpoint, the relationship between parties can have the following statuses:

- WORKING.
- COOLDOWN.
- BANNED.

The mobile app must implement a relaying mechanism to its authoritative PP. Ignoring COOLDOWN period leads to a BANNED status, which can only be reverted manually. 

A hard limit must be imposed to reduce the impact of a coordinated distributed attack with pending reservation requests. An end-user has the score of the authoritative PP. The highest trust is between a service provider and its own registered users. A good idea is to impose limits on the referred clients and reward good behavior. All trusted PP (direct relationships) will share S = h × T, where T is the total number of spots and h is a fixed value, defined by the parking lot authority, between 0.1 and 1.

$$Pen\left(pending|P_{j}\right)=P_{j}\times S\;÷\overset{n}{\underset{i=0}{\sum }}P_{i}$$

For example, as of Figure 11, P2′ and P3 will share the same limit Pen(P(P2)), as P2. Assuming P1 has T = 200, h = 0.5, and the only direct trust connection is P2 that has a probability P(P2) = 0.8, then the total pending reservation requests equals 0.8 × (200 × 0.5)/0.8 = 100. If P3 is a bad actor, it will use all the reservation slots, leaving P2 and P2′ with none and triggering a cooldown that, when ignored, ends with blocking P3. As a result, P2 is incentivized to remove bad relationships to ensure good quality of service for its trust network.

This (a) provides resistance to aggressors who would create lots of new virtual identities (sybil attacks); (b) keeps previously successful direct trust relationships, even if clients of a PP are temporarily unable to consume resources; and (c) eventually chokes relationships that have deteriorated until they improve. 

## 3. Discussion 

While our proposal is based upon in-field experience of implementing smart parking solutions, and we are confident that the approach is feasible, no complete implementation of the protocol has been achieved as of the writing of this paper. The proposal can be improved by storing the user reputation or the trust score on a public blockchain. Service discovery can also benefit from a recipe similar to ENS [19] where every parking provider advertises its services

Implementing our proposal (as an open-source protocol) is only the first step, as the methods of querying data from the parking provider can be developed as a distributed application or DApp [20]. Live data and metadata can sit on a distributed ledger and reference large amounts of data (static) from IPFS. 

We found, in the literature, two smart contract-based proposals that attempted to address the concept of a unified system, “A Blockchain-based Architecture for Integrated Smart Parking Systems” [21], and “An Open Source Solution for Smart Contract-based Parking Management” [22]. Sabbir Ahmed, Soaibuzzaman et al. [21] proposed for each parking spot to trigger a blockchain transaction when the status changes. Nikolay Buldakov, Manuel Mazzara, and Salvatore Distefano et al. [22] focused on micropayments and the transparency of interactions. While theoretically sound, the proposed solutions were lacking in practical applicability. 

As of the writing of this paper, blockchain technologies are not well suited for near real-time applications, as, in case of network congestion, transactions could take hours to finalize. Other drawbacks of the smart contract-focused approach include that the EVM code can only interact with on-chain data, high operational costs, the difficulty of developing and testing, the end-user will need to have a fully synced blockchain node, no method to ban or restrict access to abusers, and the public ledger does not offer anonymity or privacy. While the usefulness of smart contracts is undeniable, the design of the system using them should take into consideration the drawbacks and optimize for low write throughput and small data sizes.

We emphasize that our proposal is better at handling high levels of interactions and can partially offer services in the case of backend failure. In addition, our parking network can function well in the situation of a split (part of the federation refuses to communicate with the other), unlike a pure blockchain implementation that can suffer forks.

As presented in Table 2, our solution was designed to support blockchain technologies without being dependent on them while being backwards compatible (HTTP Rest APIs). Blockchain-exclusive applications require a synchronized node. Mobile applications for these would depend on external services to access blockchain data and commit transactions. Thus, the single point of failure present in centralized services is merely hidden. IPFS can be integrated into mobile applications or web pages directly. Any parking provider depending on smart contracts will lose control in favor of the owner of the unified solution contract.

## 4. Conclusions

The proposed trustless federated model increases the possibility of accessing a planetary smart parking infrastructure. A consistent data model will facilitate data exchange between Parking Providers, and the protocol will act as an abstraction layer between the technology and the human interface. The usage of IPFS (InterPlanetary File System) lowers operational costs, increases service resilience, and decentralizes the network of smart parking solutions. Static data (parking description, configuration, and statistics) can be addressed by CIDs (content identifiers) and be distributed over IPFS, so that data are moved closest to users. Even if the server that published certain information is offline, other peers that have cached the content can serve it. This proposed smart parking network is resilient by design.

We used a graph model to map the trust between the members of the smart parking network. In the future, this can be replaced with a blockchain ledger—a necessary step to make the jump from a city with multiple systems to an intelligent system that enables smart cities.

Users benefit from increased privacy because of the federated identity protocol based on Zero Knowledge Cryptography. Each identity can be blockchain based in the future. Users can trust the platform and privately use third party services without changing the interface (web app, mobile app, etc.). We explained different methods to protect the services from abuse and to isolate bad actors.

The final result is a decentralized complex system with simple interactions. IPSAN (InterPlanetary Smart Parking Architecture NOW) has the possibility to improve itself with the data it generates as a network of service providers for the benefit of both shareholders and stakeholders.

## Figures and Tables

**Table 1:** International smart parking solutions [5].

Smart Parking Application	Country	Sensors/Technology Used
Park.ME	Austria, Germany	underground sensors
SmartParking	New Zealand	underground sensors, RFID
ParkMe	Japan, USA, UK, Germany, Brazil	underground sensors
ParkAssist	USA	M4 Smart sensors, LPR
SpotHero	USA	underground sensors
EasyPark	Canada	underground sensors
PaybyPhone	France	underground sensors
ParkMobile	USA	underground sensors
AppyParking	UK	magnetometer
EasyPark Group	Sweden, Denmark and so on	transactional data and crowdsourcing
Parker	USA	underground sensors, machine vision
ParkiFi	USA	magnetometer
Best Parking	USA	underground sensors
Parkopedia	USA, Germany, Sweden and so on	predictive analytics, underground sensors
SFPark	USA	underground sensors

**Table 2:** Feature comparison between different solutions.

Feature	Our Proposal	Other 1 [21]	Other 2 [22]
Unified backend API	Yes	Yes	Yes
Unified data model	Yes	Yes	Yes
Extensible data model	Yes	No	No
Self-sovereign identity [23] compatibility	Yes	No	No
Network fork resistant	Yes	No	No
Privileged nodes	No	No	Yes
Decentralized	Yes, trustless federation	Yes	Yes
Blockchain only	No, but compatible	Yes	Yes
Low operational costs	Yes	No	No
Anonymity	Yes, ZK cryptography	No	No
Service provider autonomy	Yes	No	No
User interface development autonomy	Yes	Partially	Partially
User mobility between service providers	Yes	Locked to a smart contract	Locked to a smart contract
Can services run offline	Yes	No	No
